# Sex Differences in HIV Testing — 20 PEPFAR-Supported Sub-Saharan African Countries, 2019

**DOI:** 10.15585/mmwr.mm6948a1

**Published:** 2020-12-04

**Authors:** Bakary Drammeh, Amy Medley, Helen Dale, Anindya K. De, Shane Diekman, Randy Yee, Tiffiany Aholou, Arielle Lasry, Andrew Auld, Brittney Baack, Wayne Duffus, Ebrahim Shahul, Vincent Wong, Michael Grillo, Teeb Al-Samarrai, Shabeen Ally, Mtemwa Nyangulu, MCHD, Rose Nyirenda, Jacobus Olivier, Thato Chidarikire, Nompumelelo Khanyile, Alick A. Kayange, Oscar Ernest Rwabiyago, Upendo Kategile, Jema Bisimba, Rachel A. Weber, Gertrude Ncube, Onesimo Maguwu, Ismelda Pietersen, Denis Mali, Edington Dzinotyiweyi, Lisa Nelson, Matovu John Bosco, Kwarisiima Dalsone, Madina Apolot, Samson Anangwe, Leonard K. Soo, Mary Mugambi, Andre Mbayiha, Placidie Mugwaneza, Samuel S. Malamba, Arlene Phiri, MCD, Tina Chisenga, Mary Boyd, Chanie Temesgan, Mesfin Shimelis, Teklu Weldegebreal, Mirtie Getachew, Shirish Balachandra, Ehui Eboi, Willibord Shasha, Nicole Doumatey, Didier Adjoua, Chidozie Meribe, Jerry Gwamna, Pamela Gado, Ima John-Dada, Elie Mukinda, Luc F. Kamanga Lukusa, Lucien Kalenga, Sudhir Bunga, Victoria Achyut, John Mondi, Peter Loeto, Goabaone Mogomotsi, Jenny Ledikwe, Puleng Ramphalla, Mphotleng Tlhomola, Justine K. Mirembe, Tse Nkwoh, Laura Eno, Leonard Bonono, Nely Honwana, Noela Chicuecue, Ana Simbine, Inacio Malimane, Lenhle Dube, Munamato Mirira, Phumzile Mndzebele, Alean Frawley, Yolanda Marina Rebello Cardo, Stephanie Behel

**Affiliations:** ^1^Division of Global HIV & TB, Center for Global Health, CDC; ^2^Division of Global HIV & TB, Center for Global Health, CDC Malawi; ^3^US Agency for International Development, Office of HIV/AIDS, Washington, DC; ^4^US Department of Defense, HIV/AIDS Prevention Program; ^5^Office of the US Global AIDS Coordinator and Health Diplomacy, US Department of State; ^6^Ministry of Health, Malawi; ^7^Division of Global HIV & TB, Center for Global Health, CDC South Africa; ^8^HIV Prevention Programmes, National Department of Health, Ministry of Health, South Africa; ^9^US Agency for International Development, South Africa; ^10^US Department of Defense, HIV/AIDS Program, Tanzania; ^11^Division of Global HIV & TB, Center for Global Health, CDC Tanzania; ^12^US Agency for International Development, Tanzania; ^13^Division of Global HIV & TB, Center for Global Health, CDC Zimbabwe; ^14^Ministry of Health, Zimbabwe; ^15^US Agency for International Development, Zimbabwe; ^16^Division of Global HIV & TB, Center for Global Health, CDC Namibia; ^17^US Agency for International Development, Namibia; ^18^Ministry of Health and Social Services, Namibia; ^19^Division of Global HIV & TB, Center for Global Health, CDC Uganda; ^20^Ministry of Health, Uganda; ^21^US Agency for International Development Uganda; ^22^Department of Defence, Kenya; ^23^US Agency for International Development, Kenya; ^24^Ministry of Health, Kenya; ^25^Division of Global HIV & TB, Center for Global Health, CDC Rwanda; ^26^Ministry of Health, Rwanda; ^27^Division of Global HIV & TB, Center for Global Health, CDC Rwanda; ^28^US Agency for International Development, Zambia; ^29^Ministry of Health, Zambia; ^30^Division of Global HIV & TB, Center for Global Health, CDC Zambia; ^31^Division of Global HIV & TB, Center for Global Health, CDC Ethiopia; ^32^US Agency for International Development, Ethiopia; ^33^Federal Ministry of Health, Ethiopia; ^34^Division of Global HIV & TB, Center for Global Health, CDC Côte D’Ivoire; ^35^Ministry of Health, Côte D’Ivoire; ^36^US Agency for International Development, Côte D’Ivoire; ^37^Department of Defense, Côte D’Ivoire; ^38^Division of Global HIV & TB, Center for Global Health, CDC Nigeria; ^39^US Agency for International Development, Nigeria; ^40^Federal Ministry of Health, Nigeria; ^41^Division of Global HIV & TB, Center for Global Health, CDC Democratic Republic of Congo; ^42^Ministry of Health, Democratic Republic of Congo; ^43^US Agency for International Development, Democratic Republic of Congo; ^44^Division of Global HIV & TB, Center for Global Health, CDC South Sudan; ^45^Ministry of Health, South Sudan; ^46^Division of Global HIV & TB, Center for Global Health, CDC Botswana; ^47^Ministry of Health, Botswana; ^48^International Training and Education Center, Botswana; ^49^Division of Global HIV & TB, Center for Global Health, CDC Lesotho; ^50^Ministry of Health, Lesotho; ^51^US Agency for International Development, Lesotho; ^52^Division of Global HIV & TB, Center for Global Health, CDC Cameroon; ^53^Ministry of Health, Cameroon; ^54^Division of Global HIV & TB, Center for Global Health, CDC Mozambique; ^55^Ministry of Health, Mozambique; ^56^US Agency for International Development, Mozambique; ^57^Ministry of Health, Eswatini; ^58^US Agency for International Development, Eswatini; ^59^Division of Global HIV & TB, Center for Global Health, CDC Eswatini; ^60^Division of Global HIV & TB, Center for Global Health, CDC Angola.

Despite progress toward controlling the human immunodeficiency virus (HIV) epidemic, testing gaps remain, particularly among men and young persons in sub-Saharan Africa (*1*). This observational study used routinely collected programmatic data from 20 African countries reported to the U.S. President’s Emergency Plan for AIDS Relief (PEPFAR) from October 2018 to September 2019 to assess HIV testing coverage and case finding among adults (defined as persons aged ≥15 years). Indicators included number of HIV tests conducted, number of HIV-positive test results, and percentage positivity rate. Overall, the majority of countries reported higher HIV case finding among women than among men. However, a slightly higher percentage positivity was recorded among men (4.7%) than among women (4.1%). Provider-initiated counseling and testing (PITC) in health facilities identified approximately two thirds of all new cases, but index testing had the highest percentage positivity in all countries among both sexes. Yields from voluntary counseling and testing (VCT) and mobile testing varied by sex and by country. These findings highlight the need to identify and implement the most efficient strategies for HIV case finding in these countries to close coverage gaps. Strategies might need to be tailored for men who remain underrepresented in the majority of HIV testing programs.

In 2014, the Joint United Nations Programme on AIDS (UNAIDS) launched its 90–90–90 strategy for ending the global HIV pandemic: 90% of all persons living with HIV/AIDS (PLHIV) know their status; of these, 90% are receiving antiretroviral treatment (ART); and of these, 90% are virally suppressed (*2*). PEPFAR provides guidance on reaching these targets to all its supported countries (*3*). PEPFAR also collects data on standardized indicators as part of its Monitoring, Evaluation, and Reporting system (*4*). These data are collected by facility and community sites and are reported quarterly by each country program.

Routine program data reported to PEPFAR from October 2018 to September 2019 from 20 sub-Saharan African countries and modeling data from UNAIDS were used to identify progress toward achieving the first of the three 90–90–90 goals. These countries were selected because they collectively represent the highest HIV prevalence among PEPFAR-supported countries. Indicators used included the number of HIV tests conducted among adults, the number of HIV-positive test results, and yield (or percentage positivity) defined as the number of positive test results divided by the total number of tests reported. Results for each country are presented overall and disaggregated by sex and testing strategy. Testing strategies include index testing (offering an HIV test to the partners and biologic children of PLHIV), PITC (providers recommending an HIV test as part of routine care), VCT (HIV testing at a clinic dedicated to this purpose), and mobile testing (HIV testing offered at an ad hoc location in the community [e.g., van, workplace, or school]).The standard test statistic for the difference in proportions between men and women was computed on the basis of pooled variance formulation. P-values <0.05 were considered statistically significant for each pairwise difference and were computed under the asymptomatic normality assumption (*5*). PITC was used as the reference strategy for comparisons among strategies because the highest number of new HIV cases are identified by PITC in sub-Saharan Africa (*1*). The percentage positivity for the three other strategies (index testing, VCT, and mobile testing) was compared with PITC for each country and by sex.

From October 2018 to September 2019, PEPFAR supported 60,945,355 tests that identified 2,603,560 adults with positive HIV test results (5.0% yield; Table 1). Approximately one fifth (19.9%) of all testing occurred in South Africa. More women received tests than men (women, 40,263,510; men, 20,681,845). However, yield was slightly higher among men (970,100; 4.7% yield) than among women (1,633,460; 4.1% yield). Over one half (51.6%) of all HIV-positive results among men were reported by South Africa, Tanzania, and Zambia, and approximately one third (29.2%) were reported by South Africa alone.

**TABLE 1:** Adult human immunodeficiency virus (HIV) prevalence, by sex — 20 PEPFAR-supported African countries, October 2018–September 2019*

Country	All	HIV tests conducted		All	HIV tests positive	
		No.			No. (%)	
		Men	Women		Men	Women
Rwanda	888,336	371,405	516,931	7,343 (0.8)	2,929 (0.8)	4,414 (0.9)
Eswatini	305,714	106,448	199,266	21,341 (7.0)	8,697 (8.2)	12,644 (6.3)
Botswana	278,908	119,530	159,378	14,407 (5.2)	6,105 (5.1)	8,302 (5.2)
Namibia	398,722	130,566	268,156	14,078 (3.5)	5,385 (4.1)	8,693 (3.2)
Malawi	3,741,494	1,362,235	2,379,259	122,509 (3.3)	52,870 (3.9)	69,639 (2.9)
South Africa	12,131,042	3,996,848	8,134,194	759,465 (6.3)	267,255 (6.7)	492,210 (6.1)
Zimbabwe	2,059,970	709,379	1,350,591	112,605 (5.5)	43,340 (6.1)	69,265 (5.1)
Kenya	9,325,119	3,248,359	6,076,760	168,809 (1.8)	60,515 (1.9)	108,294 (1.8)
Zambia	4,666,548	1,843,640	2,822,908	275,966 (5.9)	111,599 (6.1)	164,367 (5.8)
Lesotho	739,505	247,653	491,852	28,899 (3.9)	11,453 (4.6)	17,446 (3.5)
Uganda	4,872,644	1,858,346	3,014,298	161,742 (3.3)	60,855 (3.3)	100,887 (3.3)
Ethiopia	401,572	153,500	248,072	8,729 (2.2)	3,543 (2.3)	5,186 (2.1)
Tanzania	6,930,758	2,415,017	4,515,741	314,364 (4.5)	121,603 (5.0)	192,761 (4.3)
Cameroon	839,762	317,881	521,881	32,435 (3.9)	11,648 (3.7)	20,787 (4.0)
Mozambique	5,651,254	1,519,954	4,131,300	281,022 (5.0)	103,433 (6.8)	177,589 (4.3)
Nigeria	4,309,213	1,348,056	2,961,157	158,351(3.7)	55,834 (4.1)	102,517 (3.5)
Côte d’Ivoire	2,200,382	564,945	1,635,437	60,058 (2.7)	19,713 (3.5)	40,345 (2.5)
DRC	811,233	235,984	575,249	41,898 (5.2)	16,062 (6.8)	25,836 (4.5)
Angola	141,292	49,215	92,077	9,208 (6.5)	3,253 (6.6)	5,955 (6.5)
South Sudan	251,887	82,884	169,003	10,331 (4.1)	4,008 (4.8)	6,323 (3.7)
**Total**	**60,945,355**	**20,681,845**	**40,263,510**	**2,603,560 (5.0)**	**970,100 (4.7)**	**1,633,460 (4.1)**

**Source:** PEPFAR Monitoring, Evaluation, and Reporting data for Accountability, Transparency, and Impact Monitoring database, October 2018–September 2019.

**Abbreviations**: DRC = Democratic Republic of Congo; PEPFAR = U.S. President’s Emergency Plan for AIDS Relief.

* Nine of the 20 countries account for 90% of HIV prevalence: Kenya, Malawi, Mozambique, Nigeria, South Africa, Tanzania, Uganda, Zambia, and Zimbabwe. Six countries have achieved the first 90 target (Botswana, Eswatini, Kenya, Lesotho, Namibia, and South Africa), and knowledge of HIV status is <70% in Angola (69%), DRC (49%), and South Sudan (24%).

Across the 19 countries (excluding Malawi because of limited data), PITC identified the most PLHIV (63.2%) compared with index testing (17.4%), VCT (11.0%), and mobile testing (8.4%). HIV case finding among men followed a similar pattern: PITC identified 57.7% of HIV-positive men, followed by index testing (21.4%), VCT (11.9%), and mobile testing (9.0%). Six countries accounted for 80.2% of HIV-positive men identified through PITC: South Africa (220,940), Mozambique (56,960), Tanzania (52,574), Zambia (38,991), Kenya, (28,814), and Uganda (26,421). Additional data for new HIV cases identified across the four HIV testing strategies both overall and by sex are provided (Table 2).

**TABLE 2:** Number and percentage of human immunodeficiency virus (HIV)–positive adults Identified, by four HIV-testing strategies, overall and by sex — 20 PEPFAR-supported countries, October 2018–September 2019*

Country	Index testing			Provider-initiated testing			Voluntary counseling and testing			Mobile testing		
	All	Men	Women	All	Men	Women	All	Men	Women	All	Men	Women
	No./Total no.^† ^(%)^§^	% Positive	% Positive	No./Total no. (%)	% Positive	% Positive	No./Total no. (%)	% Positive	% Positive	No./total no. (%)	% Positive	% Positive
Rwanda	1.4/26.6 (5.1)	4.6	5.8	2.4/545.9 (0.5)	0.5	0.4	2.6/259.9 (1.0)	0.8	1.2	0.9/55.9 (1.7)	1.0	2.8
Eswatini	3.5/13.9 (25.5)	25.6	25.4	11.7/214.9 (5.5)	6.6	5.0**^¶^**	4.0/46.6 (8.5)	7.9	9.1	2.1/30.3 (6.9)	6.5	7.2
Botswana	2.3/16.9 (13.8)	14.6	12.9	9.8/187.5 (5.3)	5.2	5.3	0.5/17.3 (3.0)	2.6	3.3	1.7/57.1 (3.0)	2.6	3.4
Namibia**	2.6/16.8 (15.6)	15.7	15.5	10.1/347.8 (2.9)	3.3	2.7	1.3/32.6 (3.9)	3.8	4.0	0.07/1.5 (4.5)	4.4	4.6
Malawi**^††^**	8.9/29.8 (30.1)	30.5	29.6	29.7/1,025.5 (2.9)	3.4	2.7**^¶^**	17.5/317.5 (5.5)	4.9	6.1**^¶^**	5.6/63.5 (8.9)	7.5	11.1**^¶^**
South Africa	28.5/92.4 (30.9)	30	31.7**^¶^**	647.8/10.2 (6.3)	6.9	6.0**^¶^**	2.2/26.3 (8.3)	7.3	11.2**^¶^**	80.9/1,747.6 (4.6)	4.2	4.9**^¶^**
Zimbabwe	23.8/73.8 (32.2)	34.5	30.2**^¶^**	79.6/1,904.1 (4.2)	4.4	4.1	5.0/45.5 (11.1)	9.7	11.9	4.2/36.6 (11.5)	9.4	13.5**^¶^**
Kenya	51.7/294.4 (17.6)	14.5	21.0**^¶^**	91.7/7,379.6 (1.2)	1.2	1.2	23.7/1,361.9 (1.7)	1.4	2.0**^¶^**	1.8/289.1 (0.6)	0.4	0.7
Zambia	63.6/222.2 (28.6)	26.4	30.7**^¶^**	105.1/2,884.3 (3.6)	3.8	3.6	87.3/1,455.2 (6.0)	5.6	6.3**^¶^**	19.9/104.8 (19.0)	15.7	22.0**^¶^**
Lesotho	4.2/17.3 (24.3)	25.4	23.3	20.2/597.0 (3.4)	4.3	3.0**^¶^**	0.4/7.8 (4.8)	3.3	5.7	4.1/117.4 (3.5)	2.7	4.5**^¶^**
Uganda	34.6/189.8 (18.2)	16.1	20.4**^¶^**	80.3/3,231.7 (2.5)	2.4	2.5	33.9/1,070.8 (3.2)	2.9	3.4	12.9/380.4 (3.4)	3.1	3.7
Ethiopia	2.8/26.1 (10.8)	10.5	11.1	4.3/313.6 (1.4)	1.5	1.3	0.9/40.8 (2.3)	1.8	2.4	0.7/21.0 (3.2)	2.5	3.9
Tanzania	116.5/544.0 (21.4)	19.9	22.9**^¶^**	153.9/5,192.5 (3.0)	3.2	2.9**^¶^**	5.0/103.5 (4.9)	3.8	5.8**^¶^**	38.9/1,090.6 (3.6)	3.4	3.7
Cameroon	5.3/46.3 (11.5)	9.5	14.3**^¶^**	21.4/708.1 (3.0)	2.8	3.1	4.5/66.2 (6.9)	5.1	8.3**^¶^**	1.1/19.1 (5.8)	4.5	7
Mozambique	40.7/151.3 (26.9)	24.9	28.9**^¶^**	177.4/4,540.9 (3.9)	5.7	3.4**^¶^**	54.3/863.6 (6.3)	6.1	6.4	8.6/95.5 (9.0)	7.2	11.6**^¶^**
Nigeria	25.1/103.5 (24.3)	19.5	30.6**^¶^**	79.4/3,007.8 (2.6)	3.2	2.4**^¶^**	38.1/947.0 (4.0)	3.3	4.6**^¶^**	15.8/250.9 (6.3)	6.1	6.4
Côte d’Ivoire	14.1/88.2 (16.0)	12.4	20.7**^¶^**	39.5/1,983.5 (2.0)	2.4	1.9**^¶^**	0.3/5.2 (6.6)	5.9	7.4	6.1/123.4 (4.9)	4.2	5.5
DRC	9.3/29.7 (31.4)	30.4	32.6	23.8/656.3 (3.6)	5.1	3.2**^¶^**	1.7/37.1 (4.6)	3.1	7.1**^¶^**	7.0/88.1 (8.0)	6.3	9.4**^¶^**
Angola	0.7/2.0 (37.3)	29.8	44.3**^¶^**	3.7/83.3 (4.5)	5	4.2	3.5/32.7 (10.8)	8.5	13.2**^¶^**	1.2/23.2 (5.2)	4.5	5.4
South Sudan	0.5/4.9 (11.0)	11.4	10.7	6.1/186.4 (3.3)	4.5	2.8**^¶^**	2.9/48.9 (6.0)	5.1	6.9	0.7/11.7 (6.4)	4.1	8.7
**Total**	**440.5/1,990.0 (22.1)**	**20.1**	**24.3^¶^**	**1,598.2/45,255.4 (3.5)**	**3.9**	**2.7^¶^**	**289.7/6,786.8 (4.3)**	**3.8**	**4.6^¶^**	**214.4/4,607.9 (4.7)**	**4.2**	**5.0^¶^**

**Source:** PEPFAR Monitoring, Evaluation, and Reporting data for Accountability, Transparency, and Impact Monitoring database, October 2018–September 2019.

**Abbreviations**: DRC = Democratic Republic of Congo; PEPFAR = U.S. President’s Emergency Plan for AIDS Relief.

* Breakdown by strategy for 19 countries (excluding Malawi because of limited data).

^†^ Number of HIV tests positive divided by the number of HIV tests conducted. Numbers are reported in thousands with one decimal place.

^§^ % indicates percent yield.

**^¶^** Statistically significant difference between men and women at p-value <0.05. The p-values were calculated based on z-tests for differences in binomial proportions with pooled variance.

** Community index testing program yield of 22.6% overall across adults.

**^††^** Data are from 5.5 districts (N = 28) in Malawi.

Although PITC identified more PLHIV than any other testing strategy, the percentage positivity of index testing was higher than PITC in all countries and for both sexes (Table 3).The number of PLHIV identified through index testing ranged from 500 in South Sudan (11.0% yield) to 116,500 in Tanzania (21.4% yield) (Table 2). The five countries that identified the most PLHIV through index testing were Uganda (34,585), Mozambique (40,681), Kenya (51,717), Zambia (63,587), and Tanzania (116,546), and yields varied from 17.6% (Kenya) to 28.6% (Zambia). These countries also accounted for two thirds (69.7%) of all HIV-positive men identified through index testing: Uganda (15,313), Mozambique (19,064), Kenya (22,259), Zambia (28,383), and Tanzania (52,040). The contribution of index testing to the total number of HIV-positive men identified in these five countries ranged from 18.1% (Mozambique) to 42.8% (Tanzania).

**TABLE 3:** Percentage positivity of four human immunodeficiency virus (HIV) testing strategies in identifying new HIV-positive adults, stratified by sex — 20 PEPFAR-supported countries, October 2018–September 2019*

Country	% Yield							
	Men				Women			
	PITC as reference*	Index testing^†^	VCT^§^	Mobile testing^¶^	PITC as reference	Index testing	VCT	Mobile testing
Rwanda	0.5	4.6**	0.8	1	0.4	5.8**	1.2	2.8**
Eswatini	6.6	25.6**	7.9	6.5	5	25.4**	9.1**	7.2**
Botswana	5.2	14.6**	2.6	2.6**	5.3	12.9**	3.3	3.4
Namibia	3.3	15.7**	3.8	4.4	2.7	15.5**	4.0	4.6
Malawi	3.4	30.5**	4.9**	7.6**	2.7	29.6**	6.1**	11.1**
South Africa	6.9	30.0**	7.3	4.2**	6	31.7**	11.2**	4.9**
Zimbabwe	4.4	34.5**	9.7**	9.4**	4.1	30.2**	11.9**	13.5**
Kenya	1.2	14.5**	1.4	0.4	1.2	21.0**	2.0**	0.7
Zambia	3.8	26.4**	5.6**	15.7**	3.6	30.7**	6.3**	22.0**
Lesotho	4.3	25.4**	3.3	2.7**	3	23.3**	5.7	4.5**
Uganda	2.4	16.1**	2.9**	3.1**	2.5	20.4**	3.4**	3.7**
Ethiopia	1.5	10.5**	1.8	2.5	1.3	11.1**	2.4	3.9**
Tanzania	3.2	19.9**	3.8	3.4	2.9	22.9**	5.8**	3.7**
Cameroon	2.8	9.5**	5.1**	4.5	3.1	14.3**	8.3**	7.0**
Mozambique	5.7	24.9**	6.1	7.2**	3.4	28.9**	6.4**	11.6**
Nigeria	3.2	19.5**	3.3	6.1**	2.4	30.6**	4.6**	6.4**
Côte d’Ivoire	2.4	12.4**	5.9**	4.2**	1.9	20.7**	7.4**	5.5**
DRC	5.1	30.4**	3.1	6.3	3.2	32.6**	7.1**	9.4**
Angola	5	29.8**	8.5**	4.5	4.2	44.3**	13.2**	5.4
South Sudan	4.5	11.4**	5.1	4.1	2.8	10.7**	6.9**	8.7**

Source: PEPFAR Monitoring, Evaluation, and Reporting data for Accountability, Transparency, and Impact Monitoring database, October 2018–September 2019.

**Abbreviations**: DRC = Democratic Republic of Congo; PEPFAR = U.S. President’s Emergency Plan for AIDS Relief; PITC = provider-initiated testing and counseling; VCT = voluntary counseling and testing.

* PITC for HIV testing of persons at health facilities.

^†^ Index testing of persons with new HIV diagnoses.

^§^ VCT to determine HIV status.

^¶^ Mobile testing outside health facilities.

** Statistically significant differences between PITC as reference compared with other strategies at p-values <0.05. The p-values were calculated on the basis of z-tests for differences in binomial proportions with pooled variance.

The yield for VCT among men was significantly higher than for PITC in eight countries (Angola, Cameroon, Côte d’Ivoire, Malawi, Mozambique, Uganda, Zambia, and Zimbabwe) but was lower in the Democratic Republic of Congo (DRC) (Table 3). Similarly, mobile testing had a significantly higher yield than PITC among men in Cameroon, Côte d’Ivoire, DRC, Malawi, Mozambique, Nigeria, Uganda, and Zimbabwe but a lower yield in Botswana, Lesotho, and South Africa. Among women, VCT had a significantly higher yield than PITC in all countries except Botswana, whereas mobile testing had a significantly higher yield than PITC in all but three countries (Botswana, South Africa, and Kenya).

These findings will help guide the Ministries of Health in selecting specific testing strategies to increase HIV-testing coverage. This will help identify persons living with HIV infection.

## Discussion

PITC identified the largest number of PLHIV, but index testing was more efficient at finding PLHIV as evidenced by a higher positivity rate. Several factors, including the absolute number of PLHIV identified, testing yield, cost per diagnosis, and the ability to reach persons with undiagnosed HIV infection, can help identify the best HIV-testing approach (*1*).

Although an efficient testing strategy in terms of identifying PLHIV, index testing is resource-intensive (*1*). For large yields of persons with a new HIV diagnosis to be identified, index testing requires a massive, potentially expensive, scale-up (*6*). However, Kenya, Tanzania, Mozambique, and Uganda have achieved substantial scale-up, demonstrating the feasibility of index testing in sub-Saharan Africa. Although the majority of PLHIV in sub-Saharan Africa receive their diagnosis through PITC, findings from this analysis show that index testing is identifying new PLHIV in many countries.

Screening tools that use a combination of clinical and behavioral questions to identify persons most likely to be HIV-positive can increase the percentage positivity of PITC. However, these screening tools must be validated to ensure high sensitivity and good specificity to avoid missed opportunities for diagnosis (*3*).

These findings show that men are less likely to be tested for HIV than women and represented 37% of HIV-positive results. Although women were twice as likely to be tested for HIV, approximately one third of new cases identified occurred among men. In all countries, women are routinely tested for HIV as part of antenatal testing, regardless of their clinical or behavioral risk factors. This might help explain why the number of tests conducted was higher among women compared with men, but the percent positivity rate was lower. PITC and index testing identified the most HIV-positive men, but mobile testing remains an important strategy to reach men who are unable or unwilling to attend health care facilities. Interventions that might improve coverage among men include flexible hours, male counselors, and integration of HIV testing into screening for other chronic conditions (*7*). Although not included in this analysis, HIV self-testing, directly offered within health facilities (*8*) or distributed by sex or needle-sharing partners as part of index testing (*9*), might also be an efficient method for reaching men.

The findings in this report are subject to at least three limitations. First, cost data are lacking. Second, data quality varies across countries despite PEPFAR’s monitoring and reporting guidance. Finally, PITC data include antenatal testing, which results in more testing among women. Testing antenatal women at lower risk also explains why the yield for PITC is lower for women than men, even though the yield is higher for women with the other three testing strategies. Strengths include the number of countries involved in the analysis and the volume of testing data collected by implementing partners.

As more PLHIV are identified, finding the remaining PLHIV will become more difficult and expensive because new cases will be harder to find. Multiple testing strategies, tailored to the epidemiologic context and targeted to populations with low access to HIV testing, can help reach these persons. Recency testing now allows programs to identify persons infected with HIV in the past year as well as clusters of incident cases. Immediate and intensified index testing efforts targeted to these clusters might reduce transmission and help countries achieve epidemic control (*10*).

**Summary**

**What is already known on this topic?**

Identifying persons with HIV infection and initiating treatment are critical to ending the HIV epidemic by 2030. Despite global progress, testing gaps remain, particularly for men.

**What is added by this report?**

From October 2018 to September 2019, men in 20 sub-Saharan African countries were half as likely as women to receive an HIV test, and 37% of the HIV-positive results were among men. Similar sex differences were observed across HIV testing strategies based upon percent positivity rates.

**What are the implications for public health practice?**

These results highlight provider-initiated testing and index testing as strategies that might improve HIV testing coverage and maximize the number of persons newly identified as HIV-positive in 20 African countries.
